# Exploring the long-term impacts of neonatal hypoglycemia to determine a safe threshold for glucose concentrations

**DOI:** 10.1007/s00431-025-06082-z

**Published:** 2025-03-22

**Authors:** Meena Garg, Sherin U. Devaskar

**Affiliations:** Department of Pediatrics, David Geffen School of Medicine at UCLA, Los Angeles, CA USA

**Keywords:** Glucose, Threshold, Newborn, Continuous glucose monitoring, Neurodevelopmental outcomes

## Abstract

Hypoglycemia and impaired metabolic transition are frequently observed in neonates during the first 24–48 h after birth [1, 2]. Severe (< 36 mg/dL or 2 mmol/L) and recurrent (3 or more episodes) hypoglycemia can cause neurological injury and developmental delays. The ambiguity regarding a threshold blood glucose concentration remains due to differing values proposed by various professional organizations. This poses a challenge in diagnosing neonatal hypoglycemia in addition to using a single blood glucose value, which in itself is not entirely reflective of various key molecular processes uncovered by in vitro or pre-clinical studies. The symptoms of hypoglycemia can also be present in conditions other than hypoglycemia, e.g., sepsis and polycythemia, and in many cases, hypoglycemia is clinically unrecognized. Therefore, early screening of at-risk and otherwise healthy-appearing neonates is essential. Continuous glucose monitoring and early interventions such as glucose gel, breast and formula feeding, and intravenous glucose administration are utilized to prevent long-term neurological impairments. However, the safe limits of serum glucose that will prevent neuroglycopenia and neural injury are elusive. The impact of early screening and available therapies on neurodevelopmental outcomes remains uncertain due to the absence of a robust clinical design and combining all causes of neonatal hypoglycemia without making further distinctions from other conditions. This review highlights the controversies in definitions and the most recent information on long-term neurodevelopmental outcomes that may impact the early management of NH.

*Conclusion*: Optimizing the definitions and treatment of neonatal dysglycemia is crucial for preventing hypoglycemia-related brain injury. Continuous glucose monitoring technology in neonates offers a promising approach for real-time screening and early intervention.

**Table:** 

**What is Known:** • *There is ongoing debate regarding the optimal glucose threshold for intervention and prevention of hypoglycemia-induced brain injury. This suggests brain injury may be incurred over a range rather than a single blood glucose concentration.*
**What is New:** *• Recent studies suggest that glucose concentrations between 36 mg/dL (2 mmol/L) and 47 mg/dL (2.6 mmol/L) are acceptable in asymptomatic neonates. However, neurological injury was observed in early school age with glucose values of <36 mg/dl (<2 mmol/L) and in mid-childhood of <30-36 mg/dL (<1.7 -2 mmol/L). This suggests brain injury may be incurred over a range rather than a single blood glucose concentration.* *• Continuous glucose monitoring (CGM) highlights real-time glucose measurement and glycemic lability in neonates. Its use may mitigate long-term neurologic injury by improving early recognition and treatment.*

**What is Known:**

• *There is ongoing debate regarding the optimal glucose threshold for intervention and prevention of hypoglycemia-induced brain injury. This suggests brain injury may be incurred over a range rather than a single blood glucose concentration.*

**What is New:**

*• Recent studies suggest that glucose concentrations between 36 mg/dL (2 mmol/L) and 47 mg/dL (2.6 mmol/L) are acceptable in asymptomatic neonates. However, neurological injury was observed in early school age with glucose values of <36 mg/dl (<2 mmol/L) and in mid-childhood of <30-36 mg/dL (<1.7 -2 mmol/L). This suggests brain injury may be incurred over a range rather than a single blood glucose concentration.*

*• Continuous glucose monitoring (CGM) highlights real-time glucose measurement and glycemic lability in neonates. Its use may mitigate long-term neurologic injury by improving early recognition and treatment.*

## Introduction

Glucose is the primary energy substrate for brain oxidative metabolism. At birth, the continuous fetal glucose supply from the placenta ceases, and the neonate must adapt to intermittent breast milk feedings to meet the glucose demand. Neonatal hypoglycemia (NH) is frequently experienced by term and near-term neonates who appear otherwise healthy, representing a delayed postnatal transition in metabolic adaptation. Hypoglycemia in the neonate is a common metabolic disturbance in the first 2–4 days of life.

**Fig. 1:**
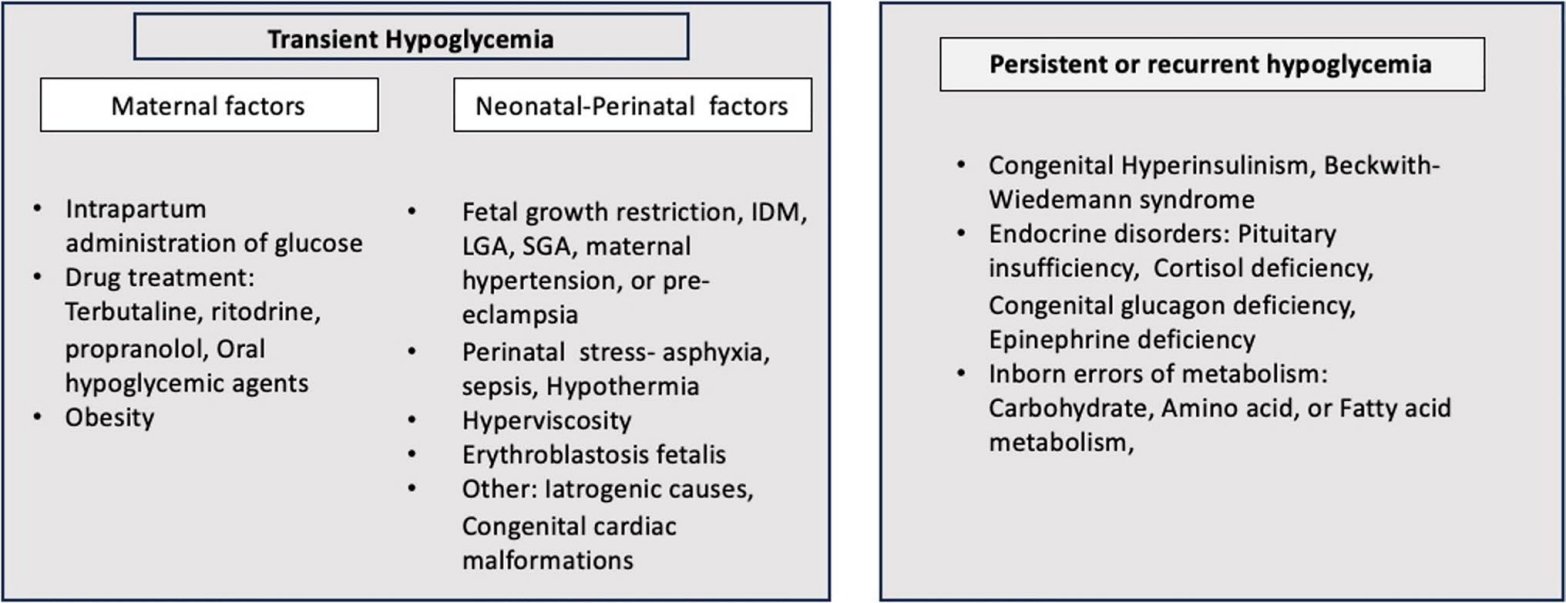
Maternal and neonatal factors affecting glucose homeostasis: differentiating transient and persistent causes of hypoglycemia

The fetal plasma glucose concentration measured in umbilical venous blood is about 80% of the maternal blood glucose values. Between 30 and 90 min after birth, plasma glucose falls immediately in all infants, stabilizing to a steady state in the next 48–72 h. Maintaining a steady glucose concentration depends on processes of glycogenolysis and gluconeogenesis versus glucose utilization and interactions between insulin and the counter-regulatory hormones glucagon, epinephrine, cortisol, and GH, with a smaller contribution from norepinephrine. Various maternal and neonatal factors are crucial for keeping newborns’ circulating glucose concentrations within the normal range, and any disruption to these factors can lead to hypoglycemia. Preterm infants, babies born large for gestational age, infants of diabetic mothers, and babies small for gestational age particularly those with intrauterine growth restriction are at high risk for NH (see Fig. 1). Around 30% of babies with associated risk factors (gestational age < 35 weeks and birth weight < 2000 g) may experience transitional hypoglycemia, and many of them may display persistent and recurrent episodes of hypoglycemia [3].

## Screening guidelines and clinical signs of neonatal hypoglycemia

Hypoglycemia is often asymptomatic, and therefore, monitoring glucose concentration in high-risk conditions is recommended (see Fig. 1). There are no universally accepted guidelines regarding the timing of the initial postnatal glucose measurement. General guidelines suggest testing glucose concentration 30 to 60 min after the first feeding and no later than 3–4 h after birth.

The clinical signs of neonatal hypoglycemia (NH) are non-specific, including jitteriness, irritability, lethargy, and poor feeding. Symptoms such as hypotonia, apnea, coma, or seizures suggest brain injury. It is essential to screen neonates at high risk for NH, as the association between clinical signs and NH is very poor. Moreover, NH may remain undetected and asymptomatic [4]. Hoermann et al. conducted a prospective cohort study to test the reliability of clinical signs and symptoms of NH by reviewing videos from infants with blood glucose concentrations of < 55 mg/dL (3 mmol/L) at less than and greater than 48 h of age. The clinical observation for signs of hypoglycemia was found to be neither sensitive nor specific in discovering NH, and there was significant interobserver variability [4].

The diagnosis of severe or recurrence of hypoglycemia may not be evident prospectively at the time of initial presentation. It is not a routine practice in nurseries to measure serum ketone concentrations in these high-risk newborn infants. Further investigations for other potential underlying causes, such as the presence of other metabolic imbalances like acidosis or alkalosis, absence of ketones, or congenital anomalies such as microcephaly, micropenis, and cleft palate, should be conducted in such cases. In instances of persistent hypoglycemia, it is recommended to conduct critical laboratory tests, including simultaneous measurement of serum insulin and glucose concentrations when blood glucose is less than 50 mg/dL, urinary ketones and organic acids, plasma cortisol, ammonia, amino acids by tandem mass spectrometry, and free and total carnitine.

### Controversies in guidelines

Defining neonatal hypoglycemia (NH) can be based on glucose concentrations falling outside postnatal statistical limits (< 2.5%) or physiological indicators, consisting of endocrine, metabolic, or neurological dysfunction as evidenced by abnormalities in sensory evoked potentials. Despite decades of reports on neurological impairment by Lucas et al. [5] and Cornblath [6], establishing an operational threshold to define NH remains challenging. Setting the operational threshold glucose concentrations is essential to ensure practical utility and wide clinical acceptance. Such a threshold requiring intervention must be higher than the concentration likely to cause organ injury. Thus therapeutic goals should be set at a concentration that is higher than the threshold. Ultimately, both glucose concentrations should provide a safety margin to prevent neurological injury [1].

Luo et al. conducted a review of ten clinical guidelines on neonatal hypoglycemia (NH), including those from the American Academy of Pediatrics (AAP), the American Academy of Breastfeeding Medicine (ABM), the Swedish National Guidelines (SNG), the Pediatric Endocrine Society (PES), and the Canadian Pediatric Society (CPS) [7]. No consensus existed among these guidelines regarding the specific blood glucose concentration at which intervention should be initiated or the target range at which glucose concentrations should be maintained [7].

Different organizations, such as the AAP, American Endocrine Society, European Pediatric Endocrine Society, British Association of Perinatal Medicine, and CPS, have differing recommendations, as shown in Table 1. Therefore, the specific definitions of NH employed in clinical practice may influence long-term outcomes.

**Table 1:** Neonatal hypoglycemia threshold: Guidelines for plasma glucose concentration needing clinical intervention based on the post-natal age as suggested by different societies and associations. The Glucose measurements were discrete and not continuous

Recommended thresholds of plasma glucose mg/dL(mmol/L)					
	0–4 h	4–24 h	24–48 h	48–72 h	72–120 h
American Academy of Pediatrics	< 40 mg/dL (2.2)	< 45 mg/dL (2.5)	< 45 mg/dL (2.5)	< 45 mg/dL (2.5)	
British Association of Perinatal Medicine	< 36 mg/dL or < 2 mmol/L (before 2nd feed)	< 36 mg/dL (< 2)	< 36 mg/dL (< 2)		
World Health Organization	< 47 mg/dL (< 2.6)	< 47 mg/dL (< 2.6)	< 47 mg/dL (< 2.6)	< 47 mg/dL (< 2.6)	
Pediatric Endocrine Society*(PES) and European Society for Pediatric Endocrinology (ESPE)	< 50 mg/dL (< 2.8)	< 50 mg/dL (< 2.8)	< 50 mg/dL (< 2.8)	< 60 mg/dL (< 3.3)	< 60 mg/dL* (< 3.3)
Canadian Pediatric Society	< 47 mg/dL (< 2.6) (at 2 h of age)	< 47 mg/dL (< 2.6)	< 47 mg/dL (< 2.6)	< 47 mg/dL (< 2.6)	

*The Glucose threshold is specific to Pediatric Endocrine Society (PES)

### Clinical definitions of neonatal hypoglycemia

Hypoglycemia in the neonatal period cannot be described by a single value. Clinicians and investigators define hypoglycemia as a plasma glucose concentration that is lower than 45 mg/dL (2.5 mmol/L) in the first 24 h of life, pre-prandial glucose lower than 50 mg/dL (2.8 mmol/L) up to 48 h of age, and lower than 60 mg/dL (3.3 mmol/L) after 48 h of age. When no risk factors for neonatal hypoglycemia are identified, it is considered transitional or a delay in postnatal metabolic adaptation that typically occurs when the glucose nadir is reached within 48–72 h, which subsequently resolves. Neuroglycopenia is a critical glucose concentration below which the supply of glucose to the brain becomes inadequate, which has not been well defined. One study noted alterations in somatosensory-evoked potentials when glucose concentration was below 47 mg/dL (2.6 mmol/L) with lower mental and motor development scores.

## Newer approach for monitoring and screening methods

**Fig. 2:**
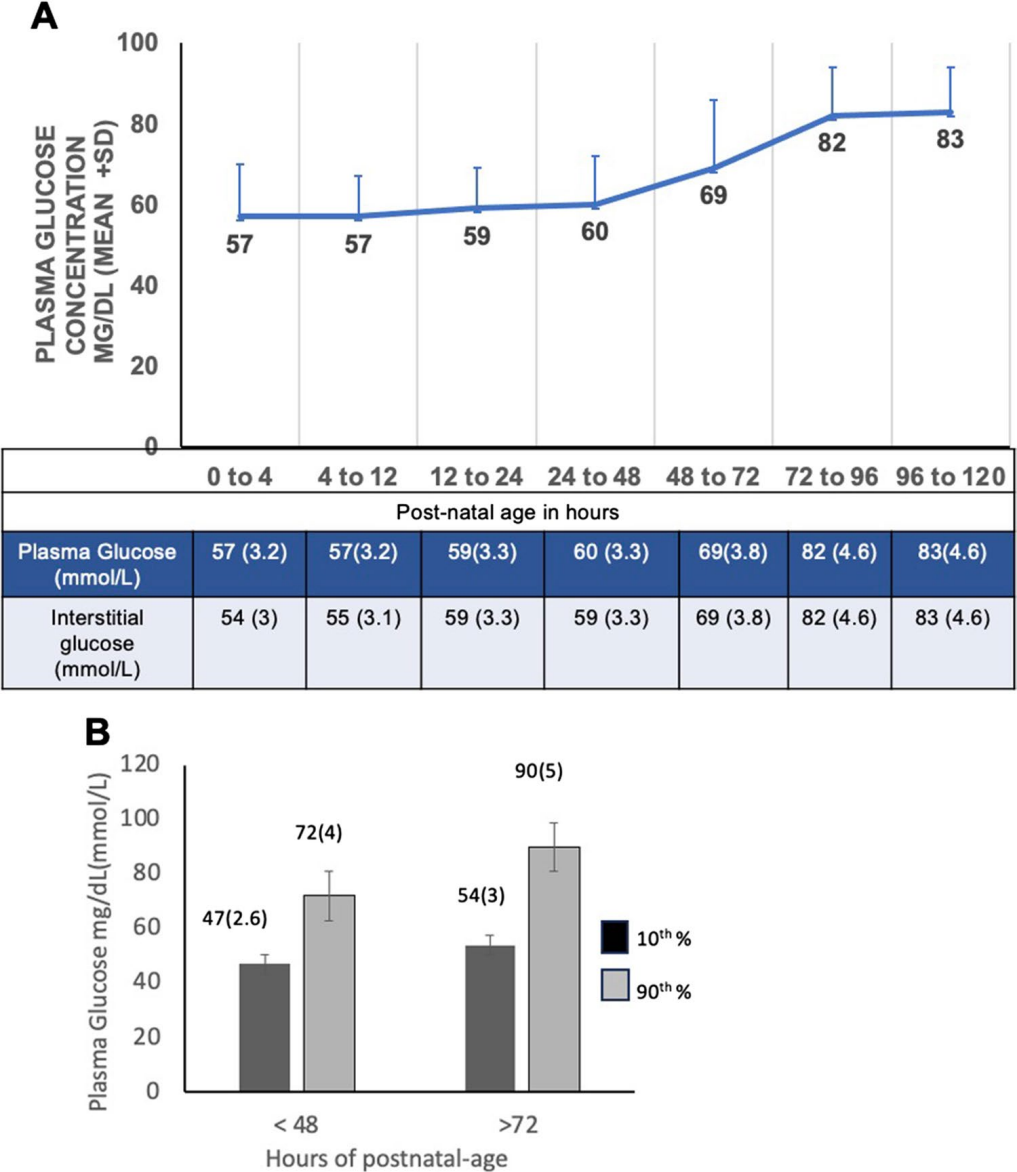
**A** Plasma glucose concentration in healthy term infants from birth to 120 hours is shown in the graph, and the table below the graph shows a comparison of plasma and interstitial glucose concentrations at the corresponding time intervals. The CGM (Medtronics, Minimed, California) was placed soon after birth to measure interstitial glucose continuously for 5 days. Plasma glucose equivalent concentrations are reported from intermittent heel stick samples obtained 60-90 minutes after birth, every 3-4 hours for the first day, and then every 12 hours for 4 days (before feeding when possible). (data from Harris et al; J Pediatr 2020;223:34-41 (GLOW study). **B** The bar graph showing 10th (in black bars) and 90th (in grey bars) percentile data for interstitial glucose derived from the continuous glucose monitoring (CGM) in Healthy Term Infants at < 48 hours and greater than 72 hours of age. (data from Harris et al; J Pediatr 2020;223:34-41 (GLOW study)

Progressive and persistent hypoglycemia can lead to brain injury, resulting in long-term adverse neurodevelopmental outcomes. The variability and non-specificity of clinical signs when they exist and the occurrence of asymptomatic hypoglycemia necessitate a laboratory measurement of serum glucose concentrations. The widely employed method for glucose determination is the rapid easy-to-deploy bedside point-of-care testing (glucose oxidase and peroxidase chromogen test strips) using a capillary blood sample. Continuous glucose monitoring (CGM) measures interstitial glucose with subcutaneously placed sensors and is increasingly utilized in clinical settings with neonates and children [3]. In recent years, CGM sensors have become smaller in size, and advanced technology has improved accuracy even at the lower end of measurements. This method has the potential to detect silent or asymptomatic hypoglycemia and thereby reduce the risk of neurodevelopmental injury. However, it is not standard of clinical care and is currently not approved for neonatal use. Additionally, cognizance is necessary regarding a discrepancy between interstitial glucose and blood glucose values due to lag in glucose transfer from the bloodstream to the interstitial fluid, along with rapidly changing glucose concentrations (Fig. 2). This was observed during the first 12 h of life in the GLOW study [2]. The 95% limits of agreement between CGM and serum measurements may be + or − 1 mmol/L or a mean absolute difference of 8.7 to 18% [8]. Therefore, CGM’s clinical application for diagnosing NH in the first 12 h of life remains an unreliable option at the present time. Newer near-infrared spectroscopy for CGM is currently being tested for use in neonates and has the potential of providing new avenues for screening in the future.

In the Glow Study [2], researchers observed trends of serum and interstitial glucose using continuous glucose monitoring (CGM) in healthy-term infants from birth to 120 h of age. The study found that plasma and interstitial glucose values increased after the first 18–24 h of post-natal age. The steady-state mean plasma glucose concentration of 59 + / − 11 mg/dL (3.3 + / − 0.6 mmol/L) was maintained for approximately 48 h, and a final steady-state concentration plateau of 89 + / − 13 mg/dL (4.9 + / − 0.7 mmol/L) was reached by the fourth day of life. The 10th percentile for the first 48 h was 47 mg/dL (2.6 mmol/L), and it was greater than 54 mg/dL (3 mmol/L) after 72 h of life. (See Figs. 2a and b).

## Management of neonatal hypoglycemia

While the priority of clinical management of NH in correcting hypoglycemia is immediate, investigation into the likely cause and vigilance regarding recurrence due to persistent hypoglycemia is of utmost importance.

### Hypoglycemia threshold and treatment

Hypoglycemia threshold during the first 48–72 h of life ranges from less than 36 mg/dL (2 mmol/L) to 50 mg/dL (2.8 mmol/dL) (refer to Table 1). These hypoglycemia thresholds do not necessarily indicate which infants will incur brain injury or develop neuroglycopenia. Many guidelines include specific treatment recommendations based on the presence or absence of symptoms of neonatal hypoglycemia (NH). See Table 1 for more details.

Neonatal hypoglycemia is diagnosed when the blood glucose falls below the threshold mentioned earlier. Hypoglycemic episodes are considered severe when blood glucose is less than 36 mg/dL (2 mmol/L) and mild when the blood glucose concentration is above 36 mg/dL (2 mmol/L) but lower than the acceptable threshold (usually less than 45 to 50 mg/dL or 2.5 to 2.8 mmol/L). Recurrent episodes of NH are considered when more than three episodes occur.

A non-inferiority trial comparing neurodevelopmental outcomes at 2 years of age found that a lower blood glucose threshold of less than 36 mg/dL (2 mmol/L) was not inferior to a higher threshold of less than 47 mg/dL (2.6 mmol/L). However, it is important to note that all the study participants were considered “healthy.” By study design, newborn infants with initial blood glucose concentrations < 36 mg/dL (2 mol/L) were excluded from this study [9]. Additionally, the concentration of glucose measured in blood samples may be lower than in the serum sample. Therefore, applying a glucose threshold of less than 36 mg/dL (2 mmol/L) in all situations may not be appropriate in preventing neurological injury.

**Fig. 3:**
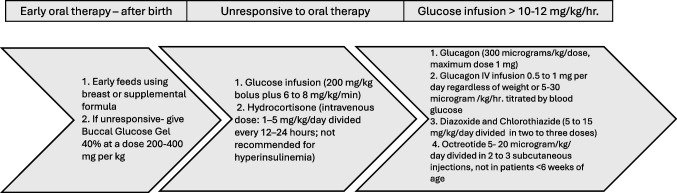
Treatment Strategies for newborn infants at high-risk of Hypoglycemia

It is crucial to promote early breastfeeding for all newborns, especially in cases of high risk for neonatal hypoglycemia (NH). During the first 48 h after birth, when NH is most common, milk supply may be low (Fig. 3). A prospective cohort study [10] found that prolonged breastfeeding for more than 30 min and feeding from both breasts was effective. If the baby’s blood glucose concentrations do not improve with breastfeeding alone, other treatment options, such as the mother’s expressed breast milk, donor breast milk, infant formula, and dextrose gel, should be considered.

#### Buccal glucose gel

The oral or buccal dextrose gel contains 40 g of dextrose per 100 mL of aqueous solution (40%). It should be administered at 200–400 mg per kg and is recommended for initial or transitional hypoglycemia unresponsive to early feeding. This initial strategy helps prevent separation of infant from the mother and improves the rate of exclusive breastfeeding after hospital discharge. Importantly, it does so without altering the rate of developmental impairment at 2 years of age, ensuring the safety of this treatment [11, 12]. Studies have shown that glucose gel should be the first line of treatment for NH since it is more effective than feeding alone and reduces the need for escalation of care in the form of NICU admissions [13, 14].

#### Intravenous glucose infusions

In infants with symptoms of NH or with severe hypoglycemia (blood glucose < 36 mg/dL) without symptoms, a rapid increase in plasma glucose is achieved by administering a bolus of 200 mg/kg (or 2 mL/kg of 10% dextrose in water) infused in less than 1 min (Fig. 3) [15]. The intravenous glucose treatment is delivered along with glucose gel and an oral feeding plan. This is followed by a continuous glucose infusion at a rate of 6 to 8 mg/kg per minute. A glucose infusion rate above 8–10 mg/kg/min strongly suggests the diagnosis of hyperinsulinism, especially when accompanied by low levels of free fatty acids (FFA) and ketones. Plasma glucose concentrations should be monitored every 1–3 h, and the infusion rate should be increased by 1 to 2 mg/kg per minute if the glucose concentration remains below 45 mg/dL (2.5 mmol/L). If the glucose concentration is between 50 and 70 mg/dL (2.8 to 3.9 mmol/L), the infusion rate should be decreased stepwise at a similar rate.

Glucagon may be effective when hepatic glycogen stores exist. Glucagon is administered when IV glucose infusion fails to stabilize the serum glucose concentrations (Fig. 3). Glucagon is a single-chain peptide primarily secreted by the alpha cells of the pancreas. Glucagon increases both glycogenolysis and gluconeogenesis, thereby raising the blood glucose concentration. The acute response by an increase in blood glucose typically occurs within 1–2 h. After administering glucagon, IV glucose infusion should be continued as the effects of glucagon are transient and short-term. Long-acting glucagon preparations, such as zinc protamine glucagon, have been used to treat glucagon deficiency states or in combination with somatostatin to address congenital hyperinsulinemia.

#### Diazoxide and chlorothiazide

Diazoxide reduces glucose-mediated insulin secretion by activating ATP-sensitive K-channels in pancreatic beta cells. It is primarily used to manage persistent hypoglycemia requiring a high glucose infusion rate, as seen in hyperinsulinemia or at times with conditions that display transient hyperinsulinism, such as intra-uterine growth restriction or a perinatal asphyxial insult. In current practice, chlorothiazide and diazoxide are routinely used together to prevent fluid overload. However, one RCT using a lower dose of diazoxide (1.5 mg/kg every 12 h) for severe or recurrent NH [16] did not demonstrate any evidence of fluid overload.

#### NH and neuroglycopenia

Glucose is the brain’s main source of energy. Brain glucose transport is facilitated by glucose transporters (GLUT1) at the blood–brain barrier so that the brain glucose concentration is about 30% of the blood glucose concentration [17] Subsequently, interstitial glucose is transported into glial cells (GLUT1) and neurons (GLUT3) via the presence of specific isoforms of the glucose transporters. The most vulnerable areas for NH injury are the thalamus, hypothalamic nuclei, prefrontal cortex, hippocampus, and amygdala, which display a higher metabolic rate with the hypothalamus exhibiting the most abundant glucose-sensing neurons (GLUT2). Neuroglycopenia has been produced by insulin-induced hypoglycemia or by mutating two major glucose transporters, namely GLUT1 at the blood–brain barrier and GLUT3 at neurons. In both cases, there is disruption of brain cellular and particularly neuronal morphology and function. Such a reduction in intracellular glucose that fuels these highly metabolically active cells results in depolarization, apoptosis, and ultimately necrosis. Supplementation with lactate or ketones does not entirely meet the cellular demand that glucose is able to provide. Thus, neuroglycopenia is responsible for mitochondrial disruption with neurons releasing glutamate, causing excitotoxicity and a propensity toward seizure activity. Neurological outcomes comprise of developmental delays, learning and behavioral problems, hyperactivity, attention difficulties, autistic traits, microcephaly, and cortical blindness. Experimental studies in rodents have shown that glucose infusion worsens neuronal death in insulin-induced hypoglycemia by increasing superoxide production, suggestive of therapeutic administration of uncontrolled glucose delivery proving to be detrimental by worsening the initial neuronal injury [18].

During NH, concentrations of lactate and BHB are suppressed in the first 24 h after birth, with BHB increasing from 24 to 72 h after birth, especially in hypoglycemic infants. These concentrations are still inadequate to support cerebral energy requirements, and they are not typically monitored in newborns with hypoglycemia, within nurseries [19].

### Long-term prognosis and neurodevelopmental outcomes

Neonatal hypoglycemia is known to pose a risk for neurologic sequelae, but there is ongoing debate about its long-term impact on neurologic consequences. More than three decades ago, Lucas et al. described motor and cognitive impairments associated with recurrent glucose concentrations below 45 mg/dL (below 2.5 mmol/L), regardless of the presence of symptoms related to neonatal hypoglycemia [5]. (Table 2).

**Table 2:** Studies reporting the Long-term Prognosis and Neurodevelopmental Outcomes of neonatal hypoglycemia ranging from 2 to 10 years of age. The blood glucose measurements were obtained intermittently and not by CGM

	Blood glucose	Study characteristics	Impairment	Age at assessment
Lucas et al. [5]	< 45 mg/dL (< 2.5 mmol/L)	Recurrent hypoglycemia	Motor and cognitive impairment	2 years
McKinlay et al. CHYLD study [20]	< 47 mg/dL (< 2.6 mmol/L)	GA < 35 weeks, BW < 2.5 kg and hypoglycemia risk	No impairment	2 years
McKinlay et al. CHYLD study [21]	< 36 mg/dL (< 2 mmol/L) or severe hypoglycemia	GA < 35 weeks, BW < 2.5 kg and hypoglycemia risk	Lower scores in executive and visual-motor functions	4.5 years
Roeper et al. [22]	< 30 mg/dL (1.7 mmol/L) severe hypoglycemia	Retrospective matched cohort	Lower IQ and cognitive function, increased deficits in fine motor function, higher attention-deficit/ hyperactivity problems	7–11 years
Wei et al. pre-hPOD study [23]	< 36 mg/dL (2 mmol/L); severe and recurrent	BW < 2.2 kg and one or more risk factors for hypoglycemia and treated with oral glucose gel	Worse visual motion perception and increased emotional-behavioral outcomes in severe recurrent hypoglycemia. No neurocognitive impairment	6–7 years
Kaiser et al. [25]	Two episodes of < 35, < 40, and < 45 mg/dL (< 1.94, < 2.2, and < 2.5 mmol/L)	Retrospective population-based cohort study, controlled for GA group (23–42 weeks), birth weight, race, sex, socioeconomic status, and education level	Lower achievement scores in literacy and mathematics proficiency at 4th grade level	10 years

McKinlay et al. conducted a study [20] with a group of 528 infants who were born with a gestational age of less than 35 weeks, a birth weight of less than 2500 g, and who had at least one risk factor for hypoglycemia. These risk factors consisted of being born to a diabetic mother, prematurity, being small or large for gestational age, or having an acute illness. Hypoglycemia was defined as having at least one blood glucose concentration lower than 47 mg/dL or 2.6 mmol/L (*n* = 216), severe hypoglycemia was defined as a blood glucose concentration that was lower than 36 mg/dL (2 mmol/L), and recurrent hypoglycemia was defined as experiencing three or more episodes of hypoglycemia. The infants were evaluated using the Bayley Scales of Infant Development III and tests for executive and visual functions. The study found no increase in neurosensory impairment at 2 years of age (Table 2). The risk of neurodevelopmental impairment remained low in the subgroup of neonates identified as having clinically undetected hypoglycemia (detected only by CGM), recurrent NH episodes, or degree of severity of hypoglycemia.

In a follow-up study conducted at 4.5 years of age, 79% (*n* = 477) of children from the original cohort underwent testing using the Weschler Preschool and Primary Scale of Intelligence, Third Edition (WPPSI-3) for full-scale IQ and verbal, performance, and processing speed subscales (mean = 100, + / − 15) [21] (Table 2). At 4.5 years of age, 216 or 53% of infants with blood glucose concentrations below 47 mg/dL showed no increased risk of neurological impairment. However, children with severe hypoglycemia (< 36 mg/dL or 2 mmol/L), recurrent hypoglycemia, or clinically undetected hypoglycemia (detected only through continuous glucose monitoring) exhibited lower scores in executive and visual-motor functions. A post hoc analysis revealed that the risk of executive and visual motor dysfunction was most significant in children with severe hypoglycemia (glucose < 36 mg/dL, 2 mmol/L) at 4.5 years of age. This longitudinal study suggests a dose-dependent effect of blood glucose concentrations on neurodevelopmental outcomes. Additionally, clinically undetected hypoglycemia identified through masked continuous glucose monitoring was associated with a four-fold increase in executive dysfunction [21].

### Mid-childhood neurodevelopmental effects of transitional hypoglycemia

In a retrospective matched cohort study, Roeper et al. studied 140 children aged 7–11 years from a database of all births at a single center (Table 2). These cohorts were matched for gestational age, birth weight, sex, and socioeconomic status. The study used standardized developmental testing to compare children with at least one episode of severe hypoglycemia (defined as blood glucose < 30 mg/dL or 1.7 mmol/L) to those with blood glucose > 30 mg/dL serving as control subjects. This threshold was chosen because a blood glucose concentration of 25 to 35 mg/dL (1.4 to 1.9 mmol/L) was a commonly suggested threshold for initiating treatment [22]. The study’s primary outcome was cognitive function, measured by a full-scale IQ test. Secondary outcomes included standardized motor, visual, and executive function scales and child behavior. The study found that the cognitive function of the hypoglycemia group was significantly lower (4.8 points) compared to the control group. Although the study was powered for the primary outcome of cognitive function, it also showed that the hypoglycemia group had an increased odds ratio of experiencing deficits in fine motor (4.9-fold) and visual motor functions (5.3-fold). Interestingly, having two or more episodes of blood glucose concentrations below 30 mg/dL (1.7 mmol/L) did not show a significant association with worse outcomes. Two or more episodes of blood glucose less than 30 mg/dL (1.7 mmol/L) were not associated with significantly worse outcomes. These results suggest that severe hypoglycemia of blood glucose < 30 mg/dL (1.7 mmol/L) results in suboptimal long-term neurodevelopmental outcomes. Since then, 25–35 mg/dL no longer serves as the threshold for initiating therapy.

The school-age follow-up of Wei et al.’s “Prevention with Oral Dextrose Study” linked poorer visual motion perception and an increased risk of emotional-behavioral difficulty to severe hypoglycemia (less than 36 mg/dL or 2 mmol/L) and recurrent hypoglycemia (more than 3 episodes) in six to seven-year-old children [23] (Table 2).

In a previous study, Rasmussen et al. [24] conducted a retrospective analysis of 71 children with NH and found no significant difference in IQ at six to nine years of age compared to 32 control subjects. However, it is important to note that this study was not sufficiently powered, and the glucose values for the control subjects were not recorded. In another large retrospective study of 1395 school-age children, Kaiser et al. reported decreased proficiency in mathematics to be associated with hypoglycemia, defined as the first two blood glucose concentrations between 40 and 45 mg/dL (2.2 and 2.5 mmol/L) [25].

## Summary

The average blood glucose concentrations show a pattern of increasing after an initial drop during the first 1–3 h after birth. However, there is significant variation in blood glucose concentrations corresponding with interstitial glucose concentrations during the first 5 days after birth. The data indicates a rise in glucose concentrations over the first 24–48 h, reaching adult values after 72 h [1]. Newborn infants follow different incremental trajectories of glucose concentration. Therefore, monitoring glucose concentrations in high-risk neonatal populations regularly is important, until stability is achieved. Although it is not a standard of care, continuous glucose monitoring (CGM) technology is advancing and awaits its future utility in clinical practice, including the detection of silent clinically unrecognized episodes of hypoglycemia.

The neurodevelopmental outcome data from carefully followed large longitudinal and matched cohort studies show that neonatal hypoglycemia has an incremental (concentration-related) adverse effect. These effects may not be identified when assessments are done at the preschool age of 2 years (CHYLD study) [19]. However, in early school years around 4 years, executive functioning becomes more advanced. In mid-childhood, after six to seven years of age, there is further advancement in executive function, fine and visual-motor functions, and emotional behavior regulation. These late childhood studies enable the clinician to recognize hypoglycemia-associated adverse neurological outcomes, at a time when neurobehavioral functioning is more advanced and necessary for the individual.

Clinicians can now take a more informed and reliable approach to defining treatment thresholds and goals for neonatal hypoglycemia. Adverse neurological outcomes have been observed in children during early school age of four to seven years [21] and 7–11 years [22, 23], indicating the importance of preventing severe hypoglycemia and maintaining blood glucose values above 36 mg/dL (2 mmol/L) in the neonatal transitional period. Severe hypoglycemia may result in adverse outcomes even after a single severe episode. Blood glucose > 47 mg/dL (2.6 mmol/L) in the first 48 h of life and > 60 mg/dL (3.3 mmol/L) after 48 h are not associated with adverse neurological outcomes and may be used as safe thresholds.

## Data Availability

No datasets were generated or analysed during the current study.

## Abbreviations

AAP: American Academy of Pediatrics
BHB: Beta-hydroxybutyrate
BPM: British Association of Perinatal Medicine
CGM: Continuous glucose monitoring
CPS: Canadian Pediatric Society
GH: Growth hormone
HIE: Hypoxic ischemic encephalopathy
NH: Neonatal hypoglycemia
PES: Pediatric Endocrine Society
WHO: World Health Organization
