# Correction to: Exploring the long-term impacts of neonatal hypoglycemia to determine a safe threshold for glucose concentrations

**DOI:** 10.1007/s00431-026-06877-8

**Published:** 2026-04-23

**Authors:** Meena Garg, Sherin U. Devaskar

**Affiliations:** Department of Pediatrics, David Geffen School of Medicine at UCLA, Los Angeles, CA USA

**Correction to: European Journal of Pediatrics (2025) 184:263**

10.1007/s00431-025-06082-z

The authors regret to inform that errors were detected in specified dosages that were identified in Figure 3 on page 5 of the originally published version of this article.

**Incorrect:** Figure 3
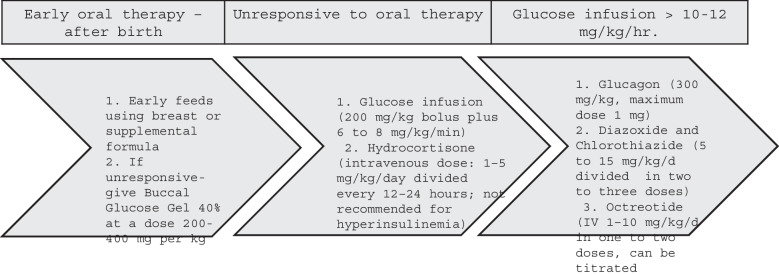


**Correct:** Figure 3
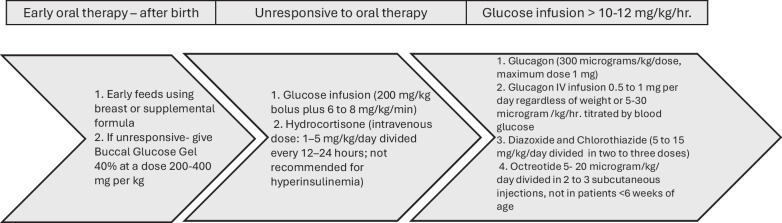


The original article has been corrected. (Comment to publisher: Please review the slides attached, second slide shows the new Figure 3 with corrected dosages for glucagon and octreotide).

